# Mammographic screening: hero or villain

**DOI:** 10.1590/1806-9282.20241501

**Published:** 2025-03-31

**Authors:** Ruffo Freitas-Junior, Aline Ferreira Bandeira de Melo Rocha, Luciano Fernandes Chala, Rafael Batista João, André Mattar

**Affiliations:** 1 Universidade Federal de Goiás, Teaching Hospital, Mastology Program – Goiânia (GO), Brazil.; 2 Goiás Anticancer Association, Araújo Jorge Hospital – Goiânia (GO), Brazil.; 3 Universidade Federal de Goiás, School of Medicine – Goiânia (GO), Brazil.; 4 Fleury Medicine and Health Group – São Paulo (SP), Brazil.; 5 Brazilian College of Radiology – São Paulo (SP), Brazil.; 6 Hospital de Caridade São Vicente de Paulo, Department of Internal Medicine – Jundiaí (SP), Brazil.; 7 Hospital da Mulher – São Paulo (SP), Brazil.; 8 Oncoclínicas – São Paulo (SP), Brazil.

## INTRODUCTION

In narratives, both real and fictional, characters and concepts often tread the fine line between heroism and its antithesis. This is the case with Felonius Gru from Despicable Me, and similarly, this concept can be applied to primary breast cancer (BC) screening examination: mammography. Both occupy the space of the "anti-hero," embodying characteristics that challenge the traditional notions of heroism.

The portrayal of Gru in Despicable Me is a classic example of an anti-hero. Initially, he is driven by ambitions, anti-social tendencies, and malevolence, plotting grand heists, such as stealing the moon^1^. However, beneath this villainous exterior lies a character filled with insecurities stemming from a childhood marked by abandonment and a longing for maternal validation. As the narrative unfolds, Gru's trajectory, influenced by the love of his three adopted daughters, sways toward redemption^2^. This complexity, oscillating between villainy and virtue, makes Gru an anti-hero par excellence. His journey exemplifies the transformative power of experiences and evolving beliefs^3^.

In medical science, mammographic screening enables the early detection of cancer, offering treatment that is generally more effective, potentially curative, less complex, and less costly^4^. Mammographic screening has proven efficacy in reducing mortality from BC^5-7^, diagnosing the disease at less advanced stages^5,8,9^, reducing the need for mastectomies, requiring less frequent axillary dissections, and leading to more limited use of chemotherapy^8,10,11^ when compared to unscreened women.

However, it is also associated with undesirable effects, such as false-positive results, overdiagnosis^12^, radiation-induced BC^13,14^, and potential psychological distress^15,16^. Due to these factors, the role of population screening has been debated^15,17^, leading to controversies regarding the age to begin and end screening and its frequency^18,19^.

While discussing mammographic screening, it is important to understand that its central idea is the identification of subclinical lesions aimed at early disease detection, thereby increasing the chances of successful treatment and consequently reducing mortality. The effectiveness of BC screening, and that of other diseases, can diminish the moment treating a clinical disease makes no difference from treating a subclinical disease^20^.

With the introduction of very effective adjuvant therapies for BC, there has been increasing speculation about the relative contribution of screening versus therapy to the observed reduction in BC mortality and whether the importance of early detection in reducing deaths due to BC will diminish as new therapies are introduced^21^. Nowadays, most national and international guidelines recommend annual or biennial mammographic screening between 40 and 74 years for average-risk populations and annual mammography or annual magnetic resonance imaging starting from a younger age for high-risk populations^19^.

Given the above, we bring a discussion on the role of mammographic screening in the female population and its position as either a hero or a villain in medical science.

## MAMMOGRAPHIC SCREENING AS A HERO

The mammographic screening has been extensively tested in numerous clinical trials^22^ and has also been evaluated in a recent meta-analysis using artificial intelligence^23^. The practice of using mammograms to detect BC early has several key benefits:

### Mortality reduction

Early detection through mammography can improve survival rates because cancers can be found when they are smaller and are more treatable, and this has been consistently shown in several different studies^21,24-28^.

Early diagnosis with the identification of early-stage tumors: Mammograms can identify BC before symptoms appear, leading to the detection of smaller tumors^12^. This early detection in turn can lead to earlier treatment, which is often less aggressive and more likely to be successful^5^.

### Decreased likelihood of needing radical surgery

When BC is caught early, there is a higher chance that a woman might undergo breast-conserving surgery (like lumpectomy) rather than more drastic measures, such as mastectomy^5^. Screening mammography has doubled the number of cases of early-stage BC that are detected each year in the United States^29^.

### Decrease of adjuvant treatment

Detecting cancer early can decrease the need for adjuvant treatment, including less invasive surgeries and the potential for avoiding chemotherapy^30^. Genomic testing, especially in early BC, helps in avoiding overtreatment and focusing on treatments that provide the actual benefit^31^.

### Cost-effectiveness

From a health economics perspective, screening can be cost-effective in terms of medical costs saved by early cancer detection and treatment, potentially leading to reduced healthcare spending on advanced cancer treatments^32,33^.

## MAMMOGRAPHIC SCREENING AS A VILLAIN

On the other hand, we have some points that can be considered problematic and can eventually disrupt patients’ lives during screening:

### Overdiagnosis

Overdiagnosis is defined as BC detected through screening that would not have become clinically apparent during a woman's lifetime with usual care. The main harm of overdiagnosis is related to the associated treatment^16,34^.

The magnitude of overdiagnosis is controversial^6^. There is an extremely wide range (0-54%) of overdiagnosis estimates related to screening^5,6,16,22^.

The rates of overdiagnosis in randomized trials were 11-22%^16^. It is projected that among women aged 50–74 years who are screened biennially, about one in every seven cases of cancer detected through screening is overdiagnosed^35^. For every BC death prevented, approximately five overdiagnosed cases will be managed^5^.

Ductal carcinomas in situ are the most common cause of overdiagnosis and should not be overtreated. Overdiagnosis is a much bigger problem in older women than in younger women, due to more competing causes of death at older ages^11^.

Despite extensive research, the measurements used to estimate overdiagnosis have some limitations, and the prospective prediction of an overdiagnosed cancer at an individual level is not possible^36^. It is impossible to know whether a specific lesion will progress and what its effect on a woman's health will be. Studies are highly heterogeneous, and estimates vary depending on the analytical approach. Until methodological standards for estimating overdiagnosis are more clearly defined, the correct estimate remains uncertain^16^.

Women who are overdiagnosed may be harmed by unnecessary procedures and treatments and by the burden of receiving a cancer diagnosis^16^. However, limiting diagnosis to symptomatic diseases is contrary to many aspects of contemporary medicine, which seeks to prevent symptomatic diseases through the successful diagnosis and treatment of pre-symptomatic conditions, even if this increases the incidence^34^. Furthermore, ongoing advances in the personalized treatment of recently diagnosed cancer cases will decrease the significance of overdiagnosis by reducing treatment morbidity^34^.

### False-positive biopsy results

The cumulative risk of undergoing fine-needle aspiration cytology, core-needle biopsy, and surgical intervention with benign results due to screening is 3.9%, 1.5%, and 0.9%, respectively^37^.

False-positive biopsy results occur when the decision to recommend a biopsy leads to a benign pathological outcome. The frequency of false-positive biopsy recommendations is 7.0-9.4% per decade of annual screening, depending on the age at the start. A woman screened annually would expect to receive a false-positive biopsy recommendation approximately once every 106-143 years of annual screening^34^.

A systematic review observed that the 10-year cumulative rates of false-positive biopsy results were higher with annual screening compared to biennial (7 vs. 5%, respectively) for women aged 40-49 years, those with dense breasts, and those using combined hormone therapy^16^.

### Radiation-induced breast cancer

Radiation-induced BC from mammographic screening and its incidence and mortality are influenced by the variability of the screening dose, subsequent diagnostic investigation, age at the start, and frequency of screening. Women with large breasts may have a higher risk of radiation-induced BC^14^.

The number of lifetime cases of radiation-induced BC and the consequent deaths reflect differences in the doses used according to the type of technology^38^. The radiation dose used depends on age, breast density, and breast thickness^13^.

For a dose of 2.5 mGy, the total lifetime risk of radiation-induced BC is 10 cases per 100,000 women if they were followed up from 50 to 85 years of age. The number of radiation-induced BC deaths is 1, and the assumed number of lives saved is approximately 350^39^.

Therefore, it can be concluded that the risk of radiation-induced BC and related mortality from mammographic screening is minimal in comparison to the number of BC deaths it prevents^13,39^.

## CONCLUSION

The ability of mammography to detect potential threats early, combined with technological advancements that refine its accuracy, underscores its irreplaceable role in modern medical science. A comprehensive understanding of mammography, including its negative and positive aspects, is essential. Recognizing its possible drawbacks while simultaneously capitalizing on its life-saving capabilities ensures the best outcomes for patients. Bringing together these narratives, the character of Gru and mammography, each in their unique way, challenge our traditional perceptions. Gru's evolution from a villain driven by egoistic ambitions to a loving father mirrors the nuanced understanding required for mammography. Both, while offering immense value, bring their inherent challenges, capturing the essence of the anti-hero.
